# Regional disparities in the flow of access to breast cancer hospitalizations in Brazil in 2004 and 2014

**DOI:** 10.1186/s12905-020-00995-7

**Published:** 2020-06-30

**Authors:** Beatriz Castro de Souza, Francisco Winter dos Santos Figueiredo, Luiz Vinicius de Alcantara Sousa, Erika da Silva Maciel, Fernando Adami

**Affiliations:** 1 Laboratório de Epidemiologia e Análise de Dados, Faculdade de Medicina do ABC – FMABC, Av. Lauro Gomes, 2000. Santo André, São Paulo, 09060-870 Brazil; 2 Universidade Federal do Tocantins, Campus Miracema. Avenida Lourdes Solino s/n°, Setor Universitário, Miracema, Tocantins Brazil

**Keywords:** Breast neoplasms, Brazil, Flow, Access

## Abstract

**Background:**

Access to the diagnosis and treatment of breast cancer in Brazil is marked by immense inequalities in the provision of specialized assistance, which leads patients to seek treatment outside the place of residence. To evaluate the variations between 2004 and 2014 in the distribution of flow between place of residence and care, and the average distance traveled for treatment of breast cancer in the administrative regions and federal states of Brazil.

**Method:**

Analysis of secondary data from the years 2004 and 2014, extracted from the Department of Informatics of the Unified Health System through the Hospital Information System. Data from Hospitalization Release Authorizations were collected, and the maps were created with TabWin 3.6 software. Descriptive analysis was performed on Stata® (StataCorp, LC) 11.0.

**Results:**

In the total flow, it was observed that there was a decrease in referrals between 2004 and 2014 in most regions. In 2004 the main direction of flow was in the Midwest and Southeast regions. In 2014, however, the intensity of these admissions was centralized in the Southeast region. In relation to the average distance traveled, the North, Northeast, and Midwest regions had the highest values of displacement. Of the 27 federative units, 17 presented an increase in average distance between these periods.

**Conclusion:**

Despite the improvement in the hospitalization of residents, in most regions and federal units, Brazilians still travel great distances when they require treatment for breast cancer.

## Background

Breast cancer has been classified as important causes of death among women and most common neoplasm present in around the world among them. Significant progression has been observed in the number of new cases of the disease in both developed and developing countries. By 2020, it is estimated that there will be a 55% increase in incidence and 58% in mortality in developing countries [1, 2].

The disparity in the distribution of specialized services stems from the unequal access to breast cancer diagnosis and treatment in Brazil, mainly in highly complex services (chemotherapy and radiotherapy). The concentration of assistance in the Southeast and South regions and the deficiency of this offer in the North region may have an impact on mortality [3–5].

The Brazilian federative political system reveals the complexity of consolidating a national health policy in an immense, unequal country “marked” by diverse organizational and environmental obstacles. Geographic disparities impose access barriers because of demographic, political, and socioeconomic conditions that transpose municipal territory [6–10].

The out-of-home treatment (TFD), established by Administrative Rule no. 55/99 of the Health Care Secretariat of the Brazilian Ministry of Health, ensures the flow of referrals to other regions when the disease is not treatable in the municipality of residence. The flows of the interstate reference of users of the Unified Health System (SUS) are regulated according to the regionalization proposal of each region [11].

The production of information on the health offer of services makes it possible to assess the geographical disparities and accessibility of health care centers. Based on this assumption and the improvements implemented in the SUS through decentralization policies in the first decade of the twenty-first century [12], it is necessary to evaluate the changes in frequency, distribution, and connections between the networks of hospital admission for breast cancer from the SUS perspective.

Therefore, the objective of this study was to evaluate the variations between 2004 and 2014 in the distribution of flows between place of residence and care, and the average distance traveled for treatment of breast cancer in the administrative regions and federal states of Brazil.

## Methods

### Study design

Secondary data analysis was performed with official data from the Brazilian Ministry of Health, obtained from the Department of Informatics of the SUS (DATASUS: www.datasus.gov.br). The units of analysis were the administrative regions (Midwest, Northeast, North, Southeast, and South) as well as federal units and the of Brazil (Acre, Alagoas, Amapá, Amazonas, Bahia, Ceará, Distrito Federal, Espírito Santo, Goiás, Maranhão, Mato Grosso, Mato Grosso do Sul, Minas Gerais, Pará, Paraíba, Paraná, Pernambuco, Piauí, Rio de Janeiro, Rio Grande do Norte, Rio Grande do Sul, Rondônia, Roraima, Santa Catarina, São Paulo, Sergipe, and Tocantins).

### Data source

Secondary data from DATASUS were extracted through the Hospital Information System of the SUS in the years 2004 and 2014. The source of the data collected was the document of Authorization of Hospitalization (AIH). “Hospitalization” refers to clinical and surgical procedures in qualified hospitals, and each AIH represents the total number of hospitalizations, not the number of patients [13].

DATASUS provides health information for states, municipalities, and the Federal District. It is a free access database and represents the main source of health information in the country [14–16], which has been used in several studies on breast cancer in Brazil [17, 18].

The tabulation tools developed by DATASUS aim to enable managers, scholars, and interested public in the area of health to obtain and analyze data from SUS information systems. These tabulators allow the selection and organization of data according to the research objective, as well as the association of the tabulations with maps, allowing visualization and spatial representation of the information [14].

The data were tabulated through the information integrator software Tab for Windows version 3.6 (TabWin: www.datasus.gov.br/tabwin), [19], developed by DATASUS. Data from the study population with diagnoses of breast cancer (C50) were selected and tabulated according to the International Classification of Diseases [20] by region and federative unit in the years 2004 and 2014.

The frequency of admissions in the selected periods and the connection of flows, tracing the displacement of users between place of residence and place of hospitalization, were analyzed with TabWin 3.6 [19]. All the connections representing in meters the rectilinear distance between the centroids of the geographic units were calculated and converted into kilometers.

### Studied variables

Variables related to flow, average distance traveled, and geographical distribution of hospital admissions for breast cancer in Brazil, stratified by administrative regions, federative units, were studied. The variables analyzed in this study are described below:

- Hospitalization by place of residence: number of breast cancer residents who registered the AIH at the place of residence;
- Local flow: percentage of hospitalizations reported at the place of residence in relation to the total number of cases reported at the place of residence;
- Routing flow: percentage of cases sent to other geographical units in relation to total cases reported in the geographical region of origin;
- Admission flow: percentage of cases admitted from other geographical units in relation to total cases reported at the place of care;
- Hospitalization by place of treatment: sum of the total number of residents remaining in the place of residence and total admissions of residents of other regions;
- Total flow: set of arrows of the flow of routing between geographic units;
- Dominant flow: main direction of referrals made from one geographic unit to another;
- Average distance traveled (km): average in kilometers of the distance that the individual traveled between the place of residence (where the first AIH was notified) and the place of treatment where the AIH was subsequently notified.

### Spatial representation

The Spatial Representation of the flows was performed with TabWin by creating maps from the table data generated by the software. The maps collected from DATASUS were selected according to geographic units for the addition of the total and dominant flow arrows.

### Descriptive analysis

The total AIH and the percentages of admission frequency by place of residence, care, and referral flows were calculated using the STATA® software (StataCorp, LC) version 11.0 from the values generated in the TabWin table. In order to evaluate the variations between flows and average distance traveled, was estimated the average difference between the years 2004 and 2014.

## Results

**Fig. 1:**
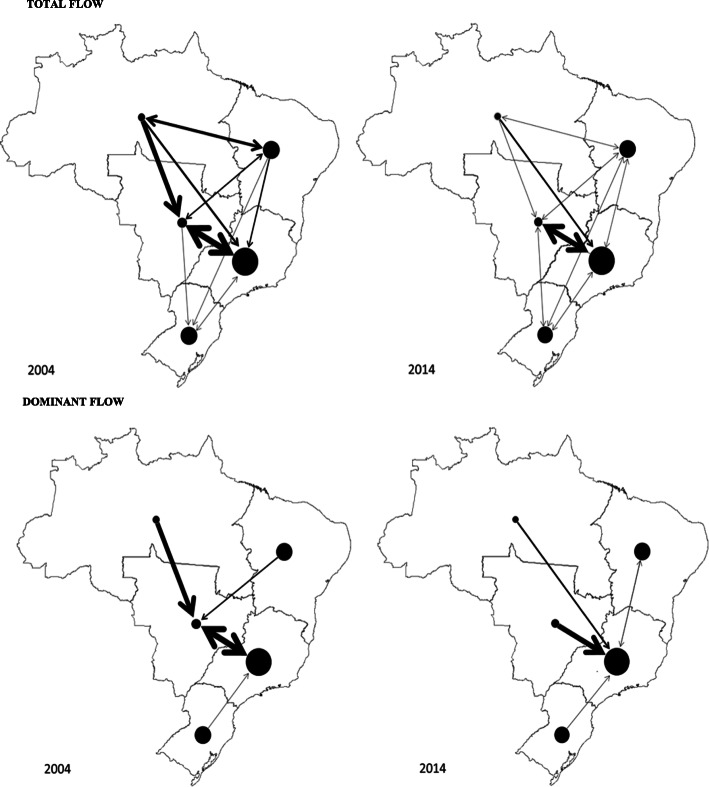
Total and dominant flow of hospital admissions for breast cancer according to region of care in 2004 and 2014. Source: Spatial representation generated using Tab Tab for Windows version 3.6 TabWin - Hospital Information System (SIH / SUS) [TabWin: www.datasus.gov.br/tabwin]

In Brazil, 36,167 AIHs were released for the population with breast cancer in 2004 and 55,965 in 2014. In the total flow (Fig. 1), there was a decrease (shown by the width of the arrows) between 2004 and 2014 in most regions, which means an improvement in the coverage of resident hospitalizations (local flow).

The main direction of flow (dominant) was for the Midwest and Southeast regions. However, in 2014, the intensity of these admissions was centralized in the Southeast, the region that admitted the most patients from other geographical units. Although the highest flow concentration was observed in these regions, in 2014 there was a greater distribution of routes (shown by the number of arrows directed between the regions) (Fig. 1). See the supplementary file - Appendix A, for more information.

In relation to this variation in flows, two administrative regions stood out: the North region with improvement of the coverage of residents’ care (local flow 3.72, reduction of the routing flow: − 3.73 and admissions flow: − 0.02 and; the Midwest region that decreased local flow: - 5.56, increased routing flow: 5.59, and; admissions flow: − 3.36 (Table 1).

**Table 1:** Variations in local, routing, and admission flow in Brazilian regions between 2004 and 2014

Regions	Variation 2004 and 2014 (%)		
	Local Flow	Routing Flow	Admissions (External Flow)
North	3.72	−3.73	−0.02
Northeast	0.05	−0.02	−0.31
Southeast	0.03	−0.08	0.73
South	−0.13	0.13	0.13
Midwest	−5.56	5.59	−3.6

Among the federative units, those that showed improvement in the range of resident hospital assistance (local flow) were Roraima (variation of 59.12), Rondônia (variation of 39.57), and Acre (variation of 16.39). Mato Grosso, Amapá, and Goiás showed a reduction of − 6.38, − 3.37, and − 2.81 in this variation, respectively (Table 2).

**Table 2:** Variations in local, routing, and admission flow in Brazilian regions and federal units between 2004 and 2014

Federative Unit	Variation 2004–2014 (%)		
	Local Flow	Routing Flow	Admissions (External Flow)
Rondônia	39.57	−39.57	2.14
Acre	16.39	−16.39	2.03
Amazonas	−2.46	2.46	3.76
Roraima	59.12	−59.12	0.00
Pará	1.08	−1.08	0.00
Amapá	−3.57	3.57	0.00
Tocantins	10.74	−10.74	−0.81
Maranhão	12.35	−12.35	0.28
Piauí	0.08	−0.08	−19.23
Ceará	0.48	−0.48	0.57
Rio Grande do Norte	−0.16	0.16	−1.98
Paraíba	2.46	−1.99	−1.99
Pernambuco	0.82	−0.82	0.69
Alagoas	−1.11	1.11	0.19
Sergipe	−0.57	0.57	−6.74
Bahia	−0.42	0.42	0.34
Minas Gerais	1.27	−1.27	0.34
Espírito Santo	0.16	−0.16	0.44
Rio de Janeiro	−0.34	0.34	−0.01
São Paulo	−0.06	0.06	1.72
Paraná	−0.67	0.67	0.92
Santa Catarina	−0.94	0.94	0.86
Rio Grande do Sul	−0.16	0.16	−0.03
Mato Grosso do Sul	1.94	−1.94	−0.04
Mato Grosso	−6.38	6.38	−1.69
Goiás	−2.81	2.81	−9.47
Distrito Federal	−0.74	0.74	3.47

**Fig. 2:**
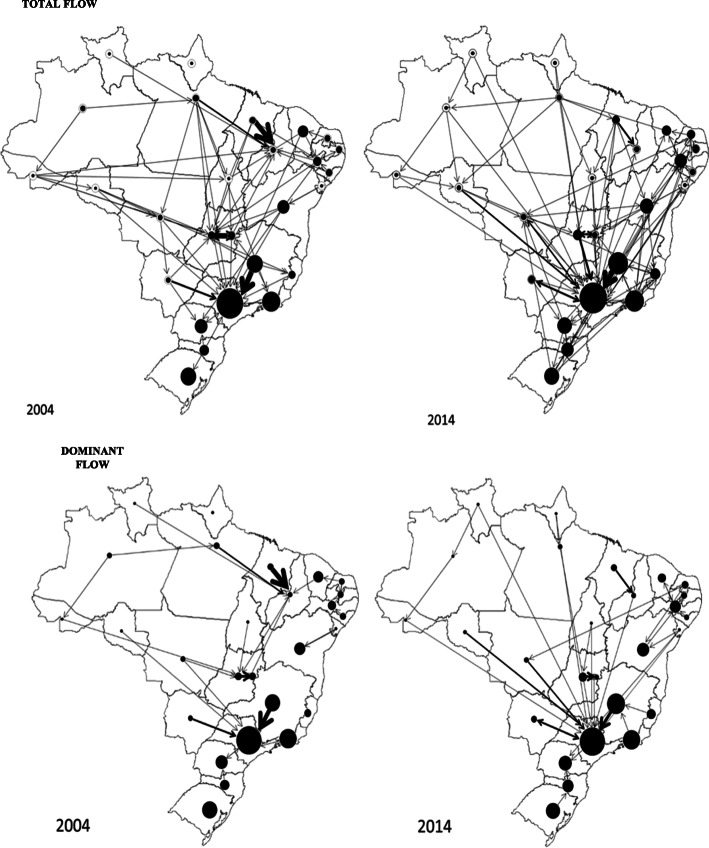
Total and dominant flow of hospital admissions for breast cancer according to the federative unit of care in 2004 and 2014. Source: Spatial representation generated using Tab Tab for Windows version 3.6 TabWin - Hospital Information System (SIH / SUS) [TabWin: www.datasus.gov.br/tabwin]

Admissions in 2004 were centralized in the federal units of São Paulo, Federal District, and Piauí, and in 2014, they were centralized in the state of São Paulo, illustrated by the number and width of the arrows (Fig. 2). See the supplementary file - Appendix B, for more information.

**Fig. 3:**
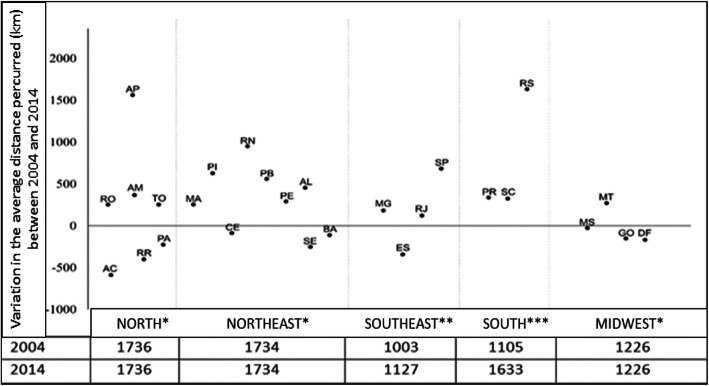
Variations in the average distance travelled (km) for hospital admission for breast cancer between the federative units and regions of Brazil.Variations in the average distance (km) between regions: *(0); **(123.86); ***(528.24). Acre (AC), Alagoas (AL), Amapá (AP), Amazonas (AM), Bahia (BA), Ceará (CE), Distrito Federal (DF), Espírito Santo (ES), Goiás (GO), Maranhão (MA), Mato Grosso (MT), Mato Grosso do Sul (MS), Minas Gerais (MG), Pará (PA), Paraíba (PB), Paraná (PR); Pernambuco (PE), Piauí (PI), Rio de Janeiro (RJ), Rio Grande do Norte (RN), Rio Grande do Sul (RS), Rondônia (RO), Roraima (RR), Santa Catarina (SC), São Paulo (SP), Sergipe (SE), Tocantins (TO)

In relation to the average distance travelled, the North, Northeast, and Midwest regions had the highest displacement values in 2004 and 2014, without distance variation (Fig. 3). Of the 27 federative units, 17 showed an increase in average distance travelled, with the highest variations observed in Amapá and Rio Grande do Sul (approximately 1500 km). Ten federative units demonstrated reduced average distance travelled, the largest reduction being observed in Acre (approximately 500 km) and Roraima (approximately 450 km) (Fig. 3).

## Discussion

When evaluating the variations between 2004 and 2014 in the distribution of flows between place of residence and care, and the average distance covered for hospitalization for breast cancer in the administrative regions and federal states of Brazil, it was observed that:

I). There was an improvement in the hospitalizations of residents (local flow) in most regions and federative units;
II). The centralization of flows led to the Southeast region, specifically in the federal unit of São Paulo;
III). There was a decrease in the hospitalizations of residents (local flow) in the Midwest region;
IV). The North region maintained the largest average distance traveled, with no variation and improvement in the local flow.
V). The federative units of the North region (Acre and Roraima) saw a decrease in the average distance traveled with improvement in the local flow.

The mapping of the flow and networks of hospitalizations for breast cancer within the SUS allowed us to measure the progression of SUS decentralization policies with their subsequent access disparities in 2004 and 2014. It is important to highlight the advances made by the SUS, established by the Federal Constitution of 1988, regarding the better distribution of resources and greater equity and access in the period studied [21–23].

Between the mid-19th and early 20th centuries, health-related policy decisions were centralized at the federal level, making it difficult to allocate resources because actual local epidemiological needs were not considered. However, with the National Health Policy/PNS-Ordinance no. 2607/GM of December 10, 2004 [24], a process of decentralization was initiated between government spheres. This was related to the own and specific demands of each geographical unit and encouraging agreement among them, which may have improved the flow of care for breast cancer in both administrative and federative regions [11, 25].

In the present study, it was observed that in 2004, dominant flow was centralized in the Midwest and Southeast regions, with the highest number of admissions for breast cancer routed by other states. However, as early as 2014, these admissions were centralized only in the Southeast region. With the centralization of flow in the Southeast region, there was a reduction in the flow of referrals to other regions. This result demonstrates the improvement in the assistance coverage of residents (local flow) in Brazil. Generally, the lower the population density, the greater the population access obstacle to health care and, consequently, the higher the per capita costs of health care [26].

In SUS health care networks, patients move from centers with less attention capacity to cities with greater capacity and complexity of services [27]. The flow of patients is organized through the displacements between reference networks (located) in large urban centers) that receive a flow of admissions from smaller cities due to their capacity and complexity of services [28, 29].

The displacement patterns of patients are modified according to the level of complexity of the treatment. Identify the poles of attraction and the fundamental relevance to identify the intensity of the users’ displacements [30, 31].

A DMP is closely correlated with accessibility to services. In a study by OLIVEIRA et al., 2004, it was found that, although the population of most municipalities closer to hospitals (the national average of the DMP index is 17.1 km), it was found that the same very small distances provoking significant reductions in the likelihood of service [28].

Therefore, the decrease in DMP can denote an improvement in the provision of health services in the treatment of breast cancer in the regions closest to the place of residence.

The increase in the number of hospitalizations of residents in the North region (local flow) between the periods studied may be related to the increase in health care coverage. In addition, all regions have routed less, with the exception of the Midwest region, which may be justified in part by the decrease in health care coverage in this region.

The Midwest Region, in 1980, presented standardized breast cancer mortality rates similar to those in the North and Northeast, but, over the years, it is approaching the magnitude of rates in the South and Southeast Regions [3]. It is noteworthy that mortality rates are strongly related to access to health services and the quality of care that is offered to women with breast cancer [5], which justifies the need to move these patients for treatment of breast cancer in other regions.

The expansion of the coverage of cancer patients care and access to cancer care is a proposal of Administrative Rule 2439/GM of December 8, 2005, which established the National Cancer Care Policy and hierarchical oncological care networks that act with reference and counter-reference flows and allowing access to health services, integral care to patients [32].

These results also corroborate the Operational Guidelines of the Referred Pact for Health of February 22, 2006, which aim to reduce social and territorial inequalities and increase access to health, as well as im prove diagnosis and local decisions to promote equity and the right to health [33].

Public policies that guarantee equal access and that reduce socioeconomic differences in different regions can overcome regional disparities in access to breast cancer diagnosis and treatment services in Brazil.

Ordinance N. 874, of May 16, 2013, institutes the National Policy for the Prevention and Control of Cancer in the Health Care Network of People with Chronic Diseases within the scope of the Unified Health System (SUS) in order to reduce the mortality; the incidence through early detection, timely treatment and palliative care.

In addition, the policy establishes that care networks are organized in a regionalized and decentralized manner, with respect to criteria of access, scale and scope [34].

In 2004, the most centralized federal units in terms of external admissions were São Paulo, Federal District, and Piauí, and in 2014 the intensity of these admissions was higher in São Paulo. In a study conducted in Brazil, from July 2005 to June 2006, more than half of the care was local, that is, performed in the municipality of residence. In terms of the volume of hospitalizations, the networks of São Paulo and Rio de Janeiro accounted for 19.4% of the total. In this same study, it was observed that, although the network for breast cancer care reaches most of the national territory, there is a lack of service provision, especially in the north of the country [29].

The North and Northeast regions had the largest displacements for out-of-home treatment in this study, possibly owing to the greater territorial distances of the country’s major reference centers (located in the Southeast). The same happened with the federative units of Amapá and Rio Grande do Sul which, located in extreme regions of the country, also presented greater average distance traveled.

As can be seen, most regions and federative units showed an increase in average distance traveled, and there was no decrease in the distance traveled to the treatment site between the studied periods.

Despite the fact that the North region presented a greater displacement for breast cancer care, the Acre and Roraima federative units were the main highlights of this study, as they demonstrated decreased average distance traveled, which characterizes a reduction of the residents’ movement to access treatment.

The delay in access to health services can influence the staging of cancer and, as a consequence, decrease the benefit of treatment, quality of life, and survival of women with breast cancer, which reflects in the mortality rates [10, 35, 36].

In regions where the delay in accessing the diagnosis and treatment of breast cancer is greater, it is more evident as high mortality rates [8, 37]. Between 2004 and 2014, there were 135,432 deaths and 475,339 hospitalizations for breast cancer in Brazilian women.

In 2014 or breast cancer was the most frequent in women in the Southeast (71.18 / 100 thousand), South (70.98 / 100 thousand) regions, Midwest (51.30 / 100 thousand) and Northeast (36.74 / 100 thousand). In the North, it was the second most incident tumor (21.29 / 100 thousand) [8, 14, 17, 18].

The trend of increasing breast cancer mortality rates, standardized by age, between 1980 and 2016, is observed in all regions of the country [1, 3]. In a study by Gonzaga et al., 2015 it shows that trends in age-adjusted breast cancer mortality rates have declined or stabilized in regions with higher socioeconomic levels and increased in places with lower socioeconomic levels [35].

The distribution of incidence by geographic region shows that the South and Southeast Regions concentrate 70% of the incidence, and the pattern of cancers is similar to that of developed countries. In the Northeast Region, even though breast cancer is more prevalent, the adjusted rates exceed the world average and are similar to the least developed regions [29, 38–41].

Thus, we can conclude that although breast cancer is more incident in the economically rich regions (South and Southeast), the highest mortality rates are in the regions with the greatest difficulty in access [6, 7, 42].

One of the limitations of this study is that hospitalizations referred only to clinical and surgical procedures and did not include high complexity procedures (chemotherapy and radiotherapy). Another limitation is that each AIH referred to the number of hospitalizations and not the number of patients.

This study highlights the importance of knowledge of the flow of access to health services. Planning for a distribution of resources should consider demographic, geographic, social, and economic aspects with a view to minimizing regional disparities in breast cancer care.

## Conclusion

Despite changes in Brazilian public health between 2004 and 2014, patients still travel long distances when they need to be treated for breast cancer. However, there has been an improvement in the hospitalization of the residents (local flow), in most regions and federative units, which represents a variation in the configuration of the care networks for hospitalization for breast cancer.

## Supplementary information

**Additional file 1: Appendix A.** Distribution, frequency and movement of hospital admissions for breast cancer between the regions and Brazil, 2004 and 2014.

**Additional file 2: Appendix B.** Distribution, frequency and movement of hospital admissions for breast cancer between the federative units of Brazil, 2004 and 2014.

## Data Availability

This study was analysis Secondary data extracted from DATASUS through the Hospital Information System of the SUS and are openly available to data collect. The authorization of Hospitalization (AIH) from the study population with diagnoses of breast cancer (C50) was tabulated through the information integrator software Tab for Windows version 3.6 [TabWin: www.datasus.gov.br/tabwin] developed and available in this platform.

## Abbreviations

TDF: The out-of-home treatment
SUS: Unified Health System
DATASUS: Department of Informatics of the SUS
AIH: Authorization of Hospitalization
TabWin: Tab for Windows version 3.6
Km: kilometers
PNS: National Health Policy
AC: Acre
AL: Alagoas
AP: Amapá
AM: Amazonas
BA: Bahia
CE: Ceará
DF: Distrito Federal
ES: Espírito Santo
GO: Goiás
MA: Maranhão
MT: Mato Grosso
MS: Mato Grosso do Sul
MG: Minas Gerais
PA: Pará
PB: Paraíba
PR: Paraná
PE: Pernambuco;
PI: Piauí
RJ: Rio de Janeiro
RN: Rio Grande do Norte
RS: Rio Grande do Sul
RO: Rondônia
RR: Roraima
SC: Santa Catarina
SP: São Paulo
SE: Sergipe
TO: Tocantins
