# Correction to: Regional disparities in the flow of access to breast cancer hospitalizations in Brazil in 2004 and 2014

**DOI:** 10.1186/s12905-020-01129-9

**Published:** 2020-12-08

**Authors:** Beatriz Castro de Souza, Francisco Winter dos Santos Figueiredo, Luiz Vinicius de Alcantara Sousa, Erika da Silva Maciel, Fernando Adami

**Affiliations:** 1 Laboratório de Epidemiologia e Análise de Dados, Faculdade de Medicina do ABC – FMABC, Av. Lauro Gomes, 2000. Santo André, São Paulo, 09060-870 Brazil; 2 Universidade Federal do Tocantins, Campus Miracema. Avenida Lourdes Solino s/n°, Setor Universitário, Miracema, Tocantins Brazil

## Correction to: BMC Women's Health (2020) 20:137 10.1186/s12905-020-00995-7

After publication of the original article [1], the authors requested the insertion of a thank you for the agreement SESACRE, UFAC and FMABC.

### **Acknowledgements**

To the Secretaria de Saúde do Acre (SESACRE), the Universidade Federal do Acre (UFAC) and Centro Universitário Saúde ABC (CUSABC), for the interinstitutional partnership through Agreement No. 007/2015, for the training of professionals in Acre, Western Amazon, Brazil.
