# Supplementary material for: Regional disparities in the flow of access to breast cancer hospitalizations in Brazil in 2004 and 2014
Source: BMC Womens Health. 2020 Jun 30;20:137. doi: 10.1186/s12905-020-00995-7 (PMC7325567; doi:10.1186/s12905-020-00995-7)
Supplement: Supplementary file 1 — Additional file 1: Appendix A. Distribution, frequency and movement of hospital admissions for breast cancer between the regions and Brazil, 2004 and 2014. [file 12905_2020_995_MOESM1_ESM.docx]

**Appendix A** Distribution, frequency and movement of hospital admissions for breast cancer between the regions and Brazil, 2004 and 2014.

| **Flow Indicators** | **Year** | | | | | |
| --- | --- | --- | --- | --- | --- | --- |
|  | **2004** | | | **2014** | | |
| **Local Flow (%): Relation between number of hospitalizations kept at the place of residence of the total number of cases reported at the place of residence.** | | | | | | |
| Regions | Hospitalization by place residence | Local Flow (n) | *Local Flow (%) | Hospitalization by place residence | Local Flow (n) | Local Flow (%) |
| North | 1238 | 1124 | 90.8 | 1495 | 1413 | 94.52 |
| Northeast | 7594 | 7558 | 99.5 | 11604 | 11552 | 99.55 |
| Southeast | 18122 | 18095 | 99.9 | 28852 | 28833 | 99.93 |
| South | 6968 | 6961 | 99.9 | 10792 | 10767 | 99.77 |
| Midwest | 2245 | 2176 | 96.9 | 3222 | 2943 | 91.34 |
| **Routing Flow (%): Relationship between number of cases sent to other regions of the total number of cases reported at the place of residence.** | | | | | | |
| Regions | Hospitalization by place residence | Cases Forwarded (n) | **Routing Flow (%) | Hospitalization by place residence | Cases Forwarded (n) | Routing Flow (%) |
| North | 1238 | 114 | 9.21 | 1495 | 82 | 5.48 |
| Northeast | 7594 | 36 | 0.47 | 11604 | 52 | 0.45 |
| Southeast | 18122 | 27 | 0.15 | 28852 | 19 | 0.07 |
| South | 6968 | 7 | 0.1 | 10792 | 25 | 0.23 |
| Midwest | 2245 | 69 | 3.07 | 3222 | 279 | 8.66 |
| **Admissions Flow (%): Relation between number of cases admitted from other regions of the total number of cases reported at the place of care.** | | | | | | |
| Regions | Hospitalization by place treatment | External Admissions (n) | ***Admissions Flows (%) | Hospitalization by place treatment | External Admissions (%) | Admissions Flow (%) |
| North | 1133 | 9 | 0.79 | 1424 | 11 | 0.77 |
| Northeast | 7593 | 35 | 0.46 | 11569 | 17 | 0.15 |
| Southeast | 18202 | 107 | 0.59 | 29220 | 387 | 1.32 |
| South | 6965 | 4 | 0.06 | 10788 | 21 | 0.19 |
| Midwest | 2274 | 98 | 4.31 | 2964 | 21 | 0.71 |

*How much of the total number of residents with cancer followed treatment in the region of residence; **How much referrals represent the total number of hospitalizations by place of residence ***How much the external admissions represent of the total of hospitalizations by place of attendance
