# Supplementary material for: Regional disparities in the flow of access to breast cancer hospitalizations in Brazil in 2004 and 2014
Source: BMC Womens Health. 2020 Jun 30;20:137. doi: 10.1186/s12905-020-00995-7 (PMC7325567; doi:10.1186/s12905-020-00995-7)
Supplement: Supplementary file 2 — Additional file 2: Appendix B. Distribution, frequency and movement of hospital admissions for breast cancer between the federative units of Brazil, 2004 and 2014. [file 12905_2020_995_MOESM2_ESM.docx]

**Appendix B** Distribution, frequency and movement of hospital admissions for breast cancer between the federative units of Brazil, 2004 and 2014.

| **Flow Indicators** | | **Year** | | | | | | | | |
| --- | --- | --- | --- | --- | --- | --- | --- | --- | --- | --- |
|  |  | **2004** | | | **2014** | | | | | |
| **Local Flow (%):**  **Relation between number of hospitalizations kept at the place of residence of the total number of cases reported at the place of residence .** | | | | | | | | | | |
| Federative Units | Hospitalization by place residence | | Local Flow (n) | *Local Flow (%) | | | Hospitalization by place residence | Local Flow (n) | | Local Flow (%) |
| RO | 58 | | 25 | 43.10 | | | 277 | 229 | | 82.67 |
| AC | 81 | | 62 | 76.54 | | | 99 | 92 | | 92.93 |
| AM | 352 | | 350 | 99.43 | | | 264 | 256 | | 96.97 |
| RR | 3 | | 1 | 33.33 | | | 53 | 49 | | 92.45 |
| PA | 551 | | 500 | 90.74 | | | 550 | 505 | | 91.82 |
| AM | 20 | | 20 | 100.0 | | | 56 | 54 | | 96.43 |
| TO | 173 | | 150 | 86.71 | | | 196 | 191 | | 97.45 |
| MA | 579 | | 439 | 75.82 | | | 803 | 708 | | 88.17 |
| PI | 394 | | 393 | 99.75 | | | 596 | 595 | | 99.83 |
| CE | 1,630 | | 1,619 | 99.33 | | | 1,575 | 1,572 | | 99.81 |
| RN | 585 | | 583 | 99.66 | | | 993 | 988 | | 99.50 |
| PB | 608 | | 590 | 97.04 | | | 826 | 818 | | 99.50 |
| PE | 1,087 | | 1,074 | 98.80 | | | 2,896 | 2,885 | | 99.62 |
| AL | 544 | | 538 | 98.90 | | | 544 | 532 | | 97.79 |
| SE | 181 | | 180 | 99.45 | | | 267 | 264 | | 98.88 |
| BA | 1,986 | | 1,952 | 98.29 | | | 3,104 | 3,038 | | 97.87 |
| MG | 3,617 | | 3,435 | 94.97 | | | 7,316 | 7,041 | | 96.24 |
| ES | 702 | | 700 | 99.72 | | | 1,607 | 1,605 | | 99.88 |
| RJ | 4,311 | | 4,305 | 99.86 | | | 6,195 | 6,165 | | 99.52 |
| SP | 9,492 | | 9,491 | 99.99 | | | 13,734 | 13,725 | | 99.93 |
| PR | 2,331 | | 2,326 | 99.79 | | | 3,870 | 3,836 | | 99.12 |
| SC | 1,359 | | 1,352 | 99.48 | | | 2,460 | 2,424 | | 98.54 |
| RS | 3,278 | | 3,278 | 100.0 | | | 4,462 | 4,455 | | 99.84 |
| MS | 333 | | 283 | 84.98 | | | 734 | 638 | | 86.92 |
| MG | 366 | | 351 | 95.90 | | | 582 | 521 | | 89.52 |
| GO | 787 | | 649 | 82.47 | | | 1,293 | 1,030 | | 79.66 |
| DF | 759 | | 756 | 99.60 | | | 613 | 606 | | 98.86 |
| **Routing Flow (%):**  **Relationship between number of cases sent to other regions of the total number of cases reported at the place of residence.** | | | | | | | | | | |
| Federative Units | Hospitalization by place of residence | | Cases Forwarded (n) | **Routing Flow (%) | | | Hospitalization by place of residence | Cases Forwarded (n) | | Routing Flow (%) |
| RO | 58 | | 33 | 56.90 | | | 277 | 48 | | 17.33 |
| AC | 81 | | 19 | 23.46 | | | 99 | 7 | | 7.07 |
| AM | 352 | | 2 | 0.57 | | | 264 | 8 | | 3.03 |
| RR | 3 | | 2 | 66.67 | | | 53 | 4 | | 7.55 |
| PA | 551 | | 51 | 9.26 | | | 550 | 45 | | 8.18 |
| AM | 20 | | 0 | 0.00 | | | 56 | 2 | | 3.57 |
| TO | 173 | | 23 | 13.29 | | | 196 | 5 | | 2.55 |
| MA | 579 | | 140 | 24.18 | | | 803 | 95 | | 11.83 |
| PI | 394 | | 1 | 0.25 | | | 596 | 1 | | 0.17 |
| CE | 1,630 | | 11 | 0.67 | | | 1,575 | 3 | | 0.19 |
| RN | 585 | | 2 | 0.34 | | | 993 | 5 | | 0.50 |
| PB | 608 | | 18 | 2.96 | | | 826 | 8 | | 0.97 |
| PE | 1,087 | | 13 | 1.20 | | | 2,896 | 11 | | 0.38 |
| AL | 544 | | 6 | 1.10 | | | 544 | 12 | | 2.21 |
| SE | 181 | | 1 | 0.55 | | | 267 | 3 | | 1.12 |
| BA | 1,986 | | 34 | 1.71 | | | 3,104 | 66 | | 2.13 |
| MG | 3,617 | | 182 | 5.03 | | | 7,316 | 275 | | 3.76 |
| ES | 702 | | 2 | 0.28 | | | 1,607 | 2 | | 0.12 |
| RJ | 4,311 | | 6 | 0.14 | | | 6,195 | 30 | | 0.48 |
| SP | 9,492 | | 1 | 0.01 | | | 13,734 | 9 | | 0.07 |
| PR | 2,331 | | 5 | 0.21 | | | 3,870 | 34 | | 0.88 |
| SC | 1,359 | | 7 | 0.52 | | | 2,460 | 36 | | 1.46 |
| RS | 3,278 | | 0 | 0.00 | | | 4,462 | 7 | | 0.16 |
| MS | 333 | | 50 | 15.02 | | | 734 | 96 | | 13.08 |
| MG | 366 | | 15 | 4.10 | | | 582 | 61 | | 10.48 |
| GO | 787 | | 138 | 17.53 | | | 1,293 | 263 | | 20.34 |
| DF | 759 | | 3 | 0.40 | | | 613 | 7 | | 1.14 |
| **Admissions Flow (%):**  **Relation between number of cases admitted from other regions of the total number of cases reported at the place of care .** | | | | | | | | | | |
| Federative Units | Hospitalization by place treatment | | Local Flow (n) | ***Admissions Flow (%) | | Hospitalization by place treatment | | External Admissions (%) | Admissions Flow (%) | |
| RO | 25 | | 0 | 0.00 | | 234 | | 5 | 2.14 | |
| AC | 64 | | 2 | 3.12 | | 97 | | 5 | 5.15 | |
| AM | 350 | | 0 | 0.00 | | 266 | | 10 | 3.76 | |
| RR | 1 | | 0 | 0.00 | | 49 | | 0 | 0.00 | |
| PA | 501 | | 1 | 0.20 | | 507 | | 1 | 0.20 | |
| AM | 20 | | 0 | 0.00 | | 54 | | 0 | 0.00 | |
| TO | 172 | | 22 | 12.79 | | 217 | | 26 | 11.98 | |
| MA | 439 | | 0 | 0.00 | | 710 | | 2 | 0.28 | |
| PI | 567 | | 174 | 30.69 | | 672 | | 77 | 11.46 | |
| CE | 1,620 | | 1 | 0.06 | | 1,582 | | 10 | 0.63 | |
| RN | 596 | | 13 | 2.18 | | 990 | | 2 | 0.20 | |
| PB | 602 | | 12 | 1.99 | | 818 | | 0 | 0.00 | |
| PE | 1,085 | | 11 | 1.01 | | 2,941 | | 50 | 1.70 | |
| AL | 538 | | 0 | 0.00 | | 533 | | 1 | 0.19 | |
| SE | 193 | | 13 | 6.74 | | 272 | | 0 | 0.00 | |
| BA | 1,953 | | 1 | 0.05 | | 3,051 | | 12 | 0.39 | |
| MG | 3,435 | | 0 | 0.00 | | 7,065 | | 24 | 0.34 | |
| ES | 706 | | 6 | 0.85 | | 1,626 | | 21 | 1.29 | |
| RJ | 4,309 | | 4 | 0.09 | | 6,173 | | 5 | 0.08 | |
| SP | 9,752 | | 261 | 2.68 | | 14,356 | | 632 | 4.40 | |
| PR | 2,334 | | 8 | 0.34 | | 3,886 | | 49 | 1.26 | |
| SC | 1,352 | | 0 | 0.00 | | 2,445 | | 21 | 0.86 | |
| RS | 3,279 | | 1 | 0.03 | | 4.457 | | 0 | 0.00 | |
| MS | 284 | | 1 | 0.35 | | 640 | | 2 | 0.31 | |
| MG | 364 | | 13 | 3.57 | | 531 | | 10 | 1.88 | |
| GO | 720 | | 71 | 9.86 | | 1,034 | | 4 | 0.39 | |
| DF | 906 | | 150 | 16.56 | | 759 | | 152 | 20.03 | |

Espírito Santo (ES), Goiás (GO), Maranhão (MA), Mato Grosso (MT), Mato Grosso do Sul (MS), Minas Gerais (MG), Pará (PA), Paraíba (PB), Paraná (PR); Pernambuco (PE), Piauí (PI), Rio de Janeiro (RJ), Rio Grande do Norte (RN), Rio Grande do Sul (RS), Rondônia (RO), Roraima (RR), Santa Catarina (SC), São Paulo (SP), Sergipe (SE), Tocantins (TO).
